# Clinical efficacy and safety of dotinurad, a novel selective urate reabsorption inhibitor, in Japanese hyperuricemic patients with or without gout: an exploratory, randomized, multicenter, double-blind, placebo-controlled, parallel-group early phase 2 study

**DOI:** 10.1007/s10157-019-01802-w

**Published:** 2019-11-21

**Authors:** Tatsuo Hosoya, Takafumi Sano, Tomomitsu Sasaki, Masahiko Fushimi, Tetsuo Ohashi

**Affiliations:** 1 Jikei University School of Medicine, 3-25-8, Nishi-Shimbashi, Minato-ku, Tokyo, 105-8461 Japan; 2 Medical R&D Division, Development Department, Fuji Yakuhin Co., Ltd., 4-383, Sakuragi-cho, Omiya-ku, Saitama-shi, 330-9508 Saitama Japan

**Keywords:** Hyperuricemia, Gout, Selective urate reabsorption inhibitor, URAT1 inhibitor, Dotinurad, FYU-981

## Abstract

**Background:**

Dotinurad, a novel selective urate reabsorption inhibitor (SURI) that has a future potential for the treatment of hyperuricemia, reduces serum uric acid levels by selectively inhibiting urate transporter 1 (URAT1). We evaluated the efficacy and safety of dotinurad in hyperuricemic Japanese patients with or without gout.

**Methods:**

The study design was an exploratory, early phase 2 study that ran for 8 weeks. It was a randomized, multicenter, double-blind, placebo-controlled, parallel-group study, and performed in a dose escalation manner. There were four study arms consisting of dotinurad 1, 2, or 4 mg, and placebo. The primary endpoint was the percent change in serum uric acid level from the baseline to the final visit. The secondary endpoint was the percentage of patients achieving a serum uric acid level ≤ 6.0 mg/dL at the final visit.

**Results:**

A total of 80 hyperuricemic patients with or without gout were enrolled and randomly assigned to the dotinurad or placebo groups. The mean percent change in serum uric acid level from the baseline to the final visit in the dotinurad 1, 2, 4 mg, and placebo groups was 37.03%, 50.91%, 64.37%, and 0.85%, respectively. The percentages of patients achieving a serum uric acid level ≤ 6.0 mg/dL at the final visit in each group were 75.0%, 89.5%, 95.2%, and none, respectively. The incidence of adverse events was comparable among all groups.

**Conclusion:**

Dotinurad has a substantial serum uric acid lowering effect in patients with hyperuricemia. No serious adverse event was found.

**ClinicalTrials.gov Identifier:**

NCT02344862

## Introduction

Hyperuricemia, defined as a serum uric acid level > 7.0 mg/dL in Japan, often causes gouty arthritis by deposition of urate crystals in the joints, tendons, and other connective tissues [1]. In recent years, the number of hyperuricemic patients with or without gout has been increasing, and the prevalence of gout in males older than 30 years is estimated to be > 1% in Japan [1].

Recent clinical reports have shown that high serum uric acid levels were associated with the increased prevalence of chronic kidney disease (CKD), hypertension, and diabetes mellitus [2–4]. Japanese guidelines for the management of hyperuricemia and gout recommend uric acid lowering therapy for hyperuricemia without gout or gouty tophi (asymptomatic hyperuricemia) especially in cases where the serum uric acid level is ≥ 8.0 mg/dL in association with lifestyle diseases, such as CKD, hypertension, and diabetes mellitus. These Japanese management guidelines advocate reducing and maintaining the serum uric acid level ≤ 6.0 mg/dL, which hopefully leads to dissolution of urate crystals in the joints [1].

Hyperuricemia is classified uric acid overproduction “overproduction type”, uric acid underexcretion “underexcretion type”, or their combination “combined type”. In Japan, prevalence of each is estimated to be 10%, 60%, and 30%, respectively [5]. The second edition of the Japanese management guidelines recommends the use of xanthine oxidase inhibitors (XOIs), such as allopurinol and febuxostat, for “overproduction type”, and uricosuric drugs, such as probenecid and benzbromarone, for “underexcretion type” [5]. In the recently revised Japanese management guidelines third edition, classification of hyperuricemia is into the three types: “underexcretion type”, “renal load type”, or “combined type”. The “renal load type”, a newly classification concept, was changed from “overproduction type” because it has been understood that the conventional “overproduction type” includes the “extrarenal underexcretion type” (decreased uric acid excretion from the intestine) [1]. In the present study, classification of hyperuricemia was implemented according to the Japanese management guidelines second edition [5], the latest version at the start of the study.

Benzbromarone is a highly potent URAT1 inhibitor that is available in a few countries, such as Japan, Germany, and the Netherlands, because it may cause serious hepatic impairment and is strongly contraindicated in patients with hepatic impairment [6]. Additionally, benzbromarone sometimes induces drug-drug interactions through strong inhibition of CYP2C9. Lately, lesinurad, a selective urate reabsorption inhibitor (SURI), has been approved in the United States and European countries. It is indicative in combination with allopurinol or febuxostat to hyperuricemic patients with gout who had not achieved target serum uric acid level with XOI monotherapy. The proposed indication was a response to renal adverse drug reactions, such as acute kidney injury and increased serum creatinine, observed with high-dose lesinurad monotherapy in a clinical study [7]. Compared to these URAT1 inhibitors, XOIs, such as allopurinol and febuxostat, are extensively used worldwide. However, allopurinol often induces severe adverse drug reactions (ADRs), such as Stevens–Johnson syndrome, toxic epidermal necrolysis, and hypersensitivity syndrome of allopurinol, and febuxostat was observed to cause hepatic impairment as an ADR [8–10]. These reports provide clinical question that there are safety-related issues associated with the XOIs and uricosuric drugs currently in use.

In most Japanese patients, hyperuricemia is classified into “underexcretion type”. Thus, treatment with hypouricemic agents that increase urinary uric acid excretion could be useful in the majority of patients. However, uricosuric drugs often are not always prescribed by clinicians despite the guidelines recommendation. One of the reasons for pharmacological properties may be derived from safety concerns and potential drug-drug interactions. Therefore, the new development of uricosuric agents having more effective and safer is anticipated.

Dotinurad, a novel SURI, reduced serum uric acid levels by selective inhibition of URAT1 in the treatment of hyperuricemia with or without gout [11]. We evaluated the efficacy and safety of dotinurad, seeking an appropriate dose of this new agent.

## Methods

### Study design

This exploratory phase 2, 8-week, randomized, multicenter, double-blind, placebo-controlled, parallel-group, dose-escalation study was performed at six clinical institutions in Japan.

### Inclusion and exclusion criteria

The inclusion criteria for this study were a serum uric acid level during the run-in period ≥ 7.0 mg/dL (patients with a history of gouty arthritis or gouty tophus), ≥ 8.0 mg/dL (patients with asymptomatic hyperuricemia who are receiving medication or had a diagnosis of hypertension, diabetes mellitus, and/or the metabolic syndrome), or ≥ 9.0 mg/dL (asymptomatic hyperuricemia without aforementioned complications), in Japanese patients aged 20–64 years on the day that written informed consent for participation in this study was obtained. The serum uric acid level criteria were followed by the Japanese guidelines [5].

The exclusion criteria were gouty arthritis that had not became asymptomatic within the 2 weeks before the day of randomization; possible disorders causing secondary hyperuricemia; hemoglobin A1c (HbA1c; NGSP) ≥ 8.4%; use of drugs that might have affected the outcome of this study during the 2 weeks before the starting day of the run-in period to randomization; hyperuricemia classified as indeterminate or “overproduction type”; complications of any serious cardiac disorder, a history of myocardial infarction, and/or an angina attack within a year; complications or a history (in the 5 years before obtaining informed consent) of cancer; complications of serious hepatic impairment (e.g., decompensated cirrhosis) or aspartate (AST) and/or alanine (ALT) aminotransferase ≥ 100 U/L; complications of a renal calculus or clinical manifestations suspicious of a urinary calculus (e.g., hematuria, back pain); estimated glomerular filtration rate (eGFR) < 60 mL/min/1.73 m^2^; blood pressure ≥ 180 mmHg systolic and/or ≥ 110 mmHg diastolic; history of drug allergy; and presence of any other clinically significant medical conditions that could potentially preclude participation in this study. If patients had been treated with any uric acid lowering drug or drugs affecting the serum uric acid level before the enrolment of this study, they were allowed to enter into this study only after a washout period of > 2 weeks.

### Treatment

**Fig. 1:**
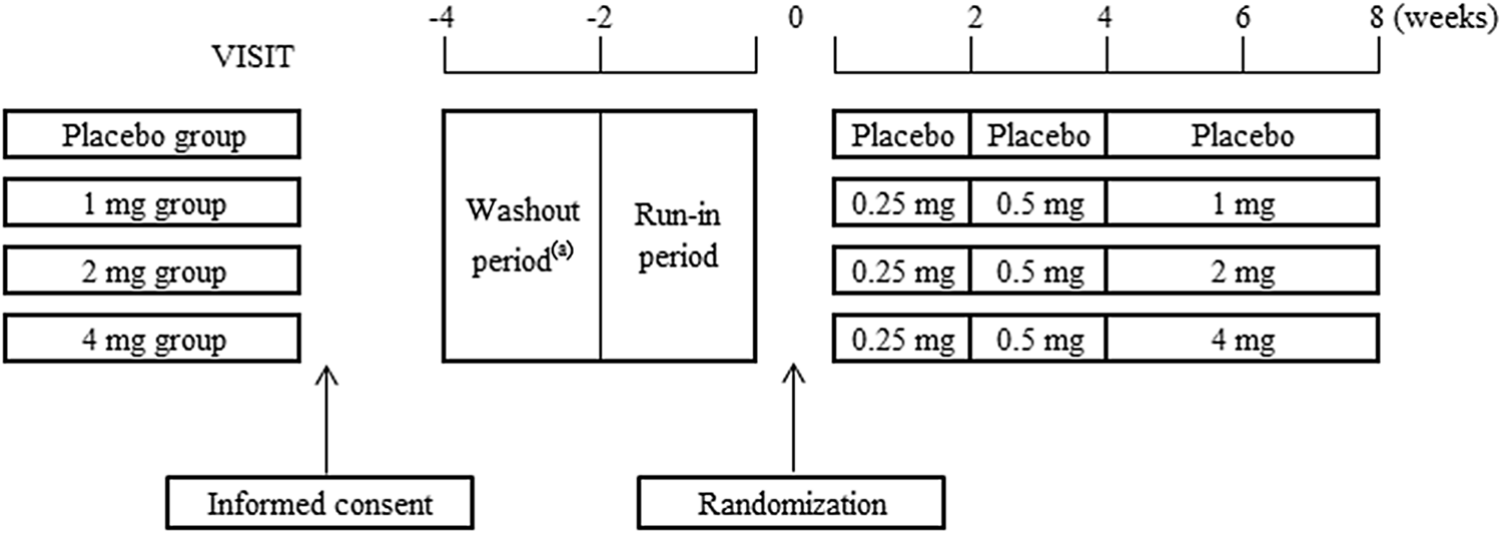
Dosing schedule. ^(a)^Patients who had been treated with uric acid lowering drugs or treatment affecting the serum uric acid level were subjected to the wash-out period

Figure 1 shows the dosing protocol. Before starting any study-related procedures, written informed consent was obtained from all the participants. At the end of the run-in period, they were randomly assigned to dotinurad 1, 2, or 4 mg, or placebo groups (ratio 1:1:1:1). A randomized block allocation of the study drug was conducted by an independent organization. After the randomization, patients were followed up including blood test every two weeks. Patients took allocated drug once daily after breakfast throughout the study. To minimize the risk of gouty arthritis in due to rapid serum uric acid reduction, we adopted the dose titration method [12]. The initial dose of dotinurad was 0.25 mg/day for the first 2 weeks and then 0.5 mg/day for 2 weeks. The maintenance dose of dotinurad was increased in a stepwise manner from the initial dose in each group (1, 2, or 4 mg/day) and treatment continued for 4 weeks until week 8 had elapsed. Furthermore, to minimize the risk of a urinary calculus in association with increased urinary uric acid excretion, investigators instructed the patients to drink enough water during the study period, and urine alkalization therapy using citrates was given together with the study drug in the following cases: (1) history of urolithiasis (2) urinary pH < 6.0 (from informed consent to end of this study), and (3) needs for the therapy at an investigator’s discretion.

To maintain the double-blind condition, the serum uric acid level was not disclosed to patients, study investigators, and local sponsor personnel from the study drug administration until the final database was disclosed.

### Classification of hyperuricemia

Hyperuricemia was classified, based on uric acid measurement in the 60-minute urine collection during the run-in period, into four types: (1) uric acid overproduction type—urinary extraction of uric acid (E_UA_)  > 0.51 mg/kg/h and uric acid clearance (C_UA_)  ≥ 7.3 mL/min/1.73 m^2^; (2) uric acid underexcretion type—E_UA_ < 0.48 or C_UA_ < 7.3; (3) combined type—E_UA_ > 0.51 and C_UA_ < 7.3; and (4) normal type—0.48 ≤ E_UA_ ≤ 0.51 and C_UA_ ≥ 7.3 [5]. The patients classified as the “uric acid overproduction type” were excluded from this study, for fear of the urinary calculus formation.

### Efficacy endpoints

The primary efficacy endpoint was the percent change in serum uric acid level from the baseline to the final visit. The secondary efficacy endpoints were the percentage of patients achieving a serum uric acid level ≤ 6.0 mg/dL at the final visit and the serum uric acid levels at each time point.

### Safety evaluations

Adverse events (AEs) and safety assessments were conducted by clinical investigators based on vital signs, 12-lead electrocardiography, clinical laboratory tests, and clinical examination throughout this study. AEs were classified according to the system organ class and preferred term (MedDRA version 17.0; Japanese Maintenance Organization, Tokyo, Japan) and were evaluated in terms of their likelihood of having a causal relationship with the study drug as well as severity, and seriousness. AEs judged to be related to the study drug were defined as ADRs.

### Statistical analyses

The primary endpoint was percent change in serum uric acid level from the baseline to the final visit, and was analyzed using the Jonckheere–Terpstra test to examine dose dependency. Based on the result of phase 1 studies, we estimated that the percent change in serum uric acid level from the baseline to the final visit of each dotinurad group (1, 2, and 4 mg groups) would be 45%, 65%, and 70%, respectively, with an assumed standard deviation (SD) of 10%. When setting the number of patients to detect the dose dependency according to a simulation with 5% level of significance and detection power of 90%, the number of patients was calculated as six patients per group. Taking into consideration the number of patients who might be excluded from the analyses and those in whom safety could be evaluated, we decided the group size to 20 patients per group.

All analyses of efficacy were evaluated using the full analysis set (FAS), which comprised all randomized patients who received at least one dose of the study drug and underwent serum uric acid measurement during at least one visit. If the serum uric acid level at the last visit (week 8) was missing, this data was compensated for by the last observation carried forward (LOCF) method. This approach was prespecified before the start of this study. In the efficacy analyses of the primary endpoint, the mean values between individual groups were compared using the Tukey–Kramer test and dose dependency was examined by the Jonckheere–Terpstra test. The Cochran–Armitage test was used to evaluate dose dependency of the secondary efficacy endpoint. The χ^2^ test was used to compare mean values between individual groups for analyses of the secondary efficacy endpoint.

Safety analyses were evaluated for the safety population (SP), which comprised all patients who took at least one dose of the study drug. The incidence of AEs was summarized as the number and proportion of patients. The Cochran–Armitage test was used to evaluate dose dependency and the χ^2^ test was used to compare incidences between individual groups.

SAS software, version 9.2 (SAS Institute, Cary, NC, USA) was used to perform statistical analyses of efficacy and safety. Unless otherwise specified, all values are expressed as mean ± SD. The statistical significance was defined based on a two-tailed *P* value of < 0.05. The statistical significance of between group differences in the patient background was defined based on a two-tailed *P* value of < 0.15.

## Results

### Patient flowcharts and baseline characteristics

**Fig. 2:**
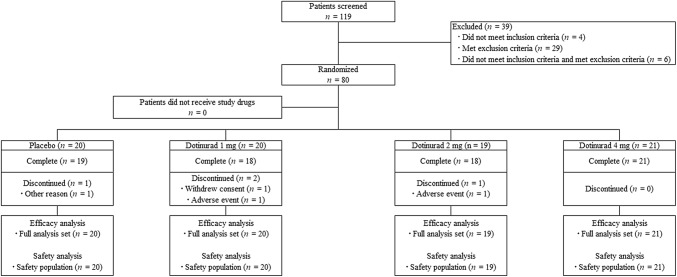
Flow diagram of study protocol

Figure 2 summarizes the flow diagram of the study protocol. Within the period of January 2014 to July 2014, 119 patients were screened, 39 were excluded, and the remaining 80 were randomized to receive dotinurad (1 mg, *n* = 20; 2 mg, *n* = 19; 4 mg, *n* = 21) or placebo (*n* = 20). Four patients did not complete the study (1 mg, one discontinued due to AE and one withdrew consent; 2 mg, one discontinued due to an AE; placebo, one met discontinuation criteria). All patients who received the allocated drug were included in the FAS and SP.

The baseline characteristics of patients were comparable among the four groups, except for eGFR (Table 1). All patients were Japanese males, aged 30–64 years, and 85.0% were classified into the “underexcretion type”, while 96.3% had a history of gouty arthritis. Mean serum uric acid level during the run-in period ranged 8.79–8.95 mg/dL in the overall groups.

**Table 1:** Baseline characteristics of patients enrolled

Characteristic	Placebo (n = 20)	Dotinurad				*P* value
		1 mg (*n* = 20)	2 mg (*n* = 19)	4 mg (*n* = 21)	Total (*n* = 60)	
Sex						
Male	20	20	19	21	60	–
Female	0	0	0	0	0	
Age (year)						
Mean ± SD	48.8 ± 9.7	52.7 ± 9.4	50.1 ± 8.3	51.2 ± 7.4	51.4 ± 8.3	0.544^a^
Height (cm)						
Mean ± SD	170.07 ± 6.44	173.03 ± 6.69	171.47 ± 6.54	172.39 ± 7.05	172.31 ± 6.69	0.532^a^
Weight (kg)						
Mean ± SD	76.23 ± 14.47	73.22 ± 10.40	75.92 ± 13.67	78.95 ± 17.36	76.08 ± 14.13	0.647^a^
Serum uric acid (mg/dL)						
Mean ± SD	8.85 ± 1.30	8.79 ± 1.00	8.91 ± 1.23	8.95 ± 1.48	8.88 ± 1.24	0.980^a^
eGFR (mL/min/1.73 m^2^)						
Mean ± SD	80.8 ± 10.5	75.8 ± 9.5	73.6 ± 11.3	74.8 ± 9.7	74.8 ± 10.0	0.139*^a^
Medical history of hyperuricemia						
Number of patients	20	20	19	21	60	0.596^b^
History of gouty arthritis						
Number of patients	19	19	19	20	58	0.808^b^
Existence of gouty tophus						
Number of patients	1	0	0	0	0	0.386^b^
Drinking habits						
Number of patients	17	15	17	19	51	0.501^b^
Classification of hyperuricemia						
Uric acid underexcretion type	15	18	17	18	53	0.519^b^
Combined or normal type	5	2	2	3	7	

eGFR for male (mL/min/1.73 m^2^) = 194 × Serum creatinine^−1.094^ × Age^−0.287^ [14]

Definition of drinking habit: consumption of alcohol more than 3 days of the week and consumption of more than 500 mL beer or 60 mL whisky in a day

*Total* total of dotinurtad

*P < 0.15

^a^ANOVA

^b^χ^2^ test

### Efficacy

One patient in the placebo group was withdrawn from this study before week 4 due to discontinuation criteria. According to the prespecified statistical missing data management criterion, the serum uric acid level data for this patient at week 2 were not adopted from the primary and secondary efficacy analyses.

### The primary efficacy endpoint

The percent changes (mean ± SD) in serum uric acid level from the baseline to the final visit in each group were 37.03 ± 8.92%, 50.91 ± 10.61%, 64.37 ± 7.67%, and 0.85 ± 14.53% in the dotinurad 1, 2, and 4 mg and placebo groups, respectively (Table 2), indicating that a dose dependency was observed (*P* < 0.001, Jonckheere–Terpstra test). Furthermore, significant percent changes were noted in each dotinurad group compared to the placebo group (*P* < 0.001, Tukey–Kramer test).

**Table 2:** Primary and secondary efficacy endpoints

End point	Category	Placebo (*n* = 19)	Dotinurad		
			1 mg (*n* = 20)	2 mg (*n* = 19)	4 mg (*n* = 21)
Percent change in serum uric acid level from the baseline to the final visit	Mean ± SD	0.85 ± 14.53	37.03 ± 8.92	50.91 ± 10.61	64.37 ± 7.67
	95% Confidence Interval	− 6.15 to 7.86	32.85 to 41.21	45.80 to 56.03	60.88 to 67.86
	Jonckheere–Terpstra test	*P* < 0.001*			
	Tukey–Kramer test	–	*P* < 0.001*	*P* < 0.001*	*P* < 0.001*
Percentage of patients with a serum uric acid level ≤ 6.0 mg/dL at the final visit	Number (%)	0 (0.0)	15 (75.0)	17 (89.5)	20 (95.2)
	95% Confidence Interval	0.0 to 17.6	50.9 to 91.3	66.9 to 98.7	76.2 to 99.9
	Cochran–Armitage test	*P* < 0.001*			
	χ^2^ test	–	*P* < 0.001*	*P* < 0.001*	*P* < 0.001*

Jonckheere–Terpstra test and Cochran–Armitage test were used to evaluate dose dependency in the groups of 1 mg, 2 mg, and 4 mg, and placebo

Tukey–Kramer test and χ^2^ test were adjusted about placebo vs. each dotinurad group

*P < 0.05

### The secondary efficacy endpoint

The percentages of patients with serum uric acid level ≤ 6.0 mg/dL at the final visit in each group were 75.0% (15/20 patients), 89.5% (17/19 patients), 95.2% (20/21 patients), and none (0/19 patients) in the dotinurad 1, 2, and 4 mg, and placebo groups, respectively (Table 2), indicating a dose dependency (*P* < 0.001, Cochran–Armitage test). Furthermore, significant differences were observed between each dotinurad group and the placebo group (*P* < 0.001, χ^2^ test).

**Fig. 3:**
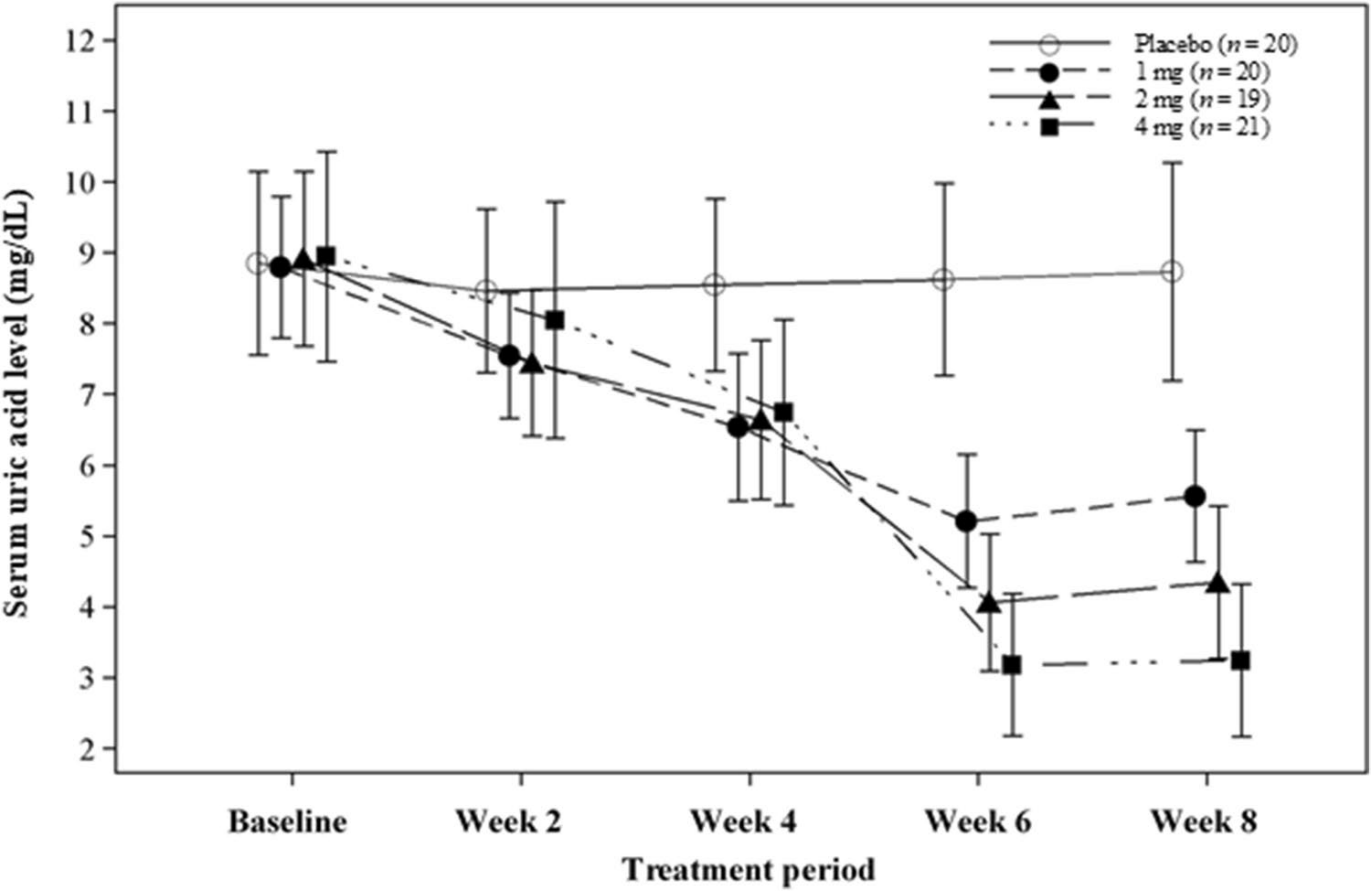
Changes in serum uric acid level in response to follow treatment with dotinurad. Error bars indicates standard deviation

Figure 3 shows the changes in serum uric acid level in response to follow treatment with dotinurad. In the dotinurad groups, decrease in serum uric acid level compared to baseline was observed and the serum uric acid lowering effect tended to be enhanced dose increased. Mean serum uric acid levels at the final visit for the dotinurad 1, 2 and 4 mg and placebo groups were 5.52, 4.36, 3.24, and 8.73 mg/dL, respectively.

### Safety

Table 3 shows the incidences of AEs and ADRs in this study. AEs occurred in six patients (30.0%), 12 patients (63.2%), 11 patients (52.4%), and 7 patients (35.0%) in the dotinurad 1, 2, and 4 mg, and placebo groups, respectively. The incidences of AEs did not increase with dose escalation (*P* = 0.089, Cochran–Armitage test); however, significant differences were observed in the groups given 1 and 2 mg (*P* = 0.038, χ^2^ test). Most AEs were mild or moderate in severity and there were no serious AEs including deaths. The AEs that leaded to discontinuation of the study included abdominal discomfort (*n* = 1) in the dotinurad 2 mg group and somnolence (*n* = 1) in the 1 mg group. The investigators regarded that the abdominal discomfort as one of the ADRs. Regarding ADRs, the incidences in each group were comparable among the groups (*P* ≥ 0.163, χ^2^ test). The incidences of ADRs was not associated with dose escalation (*P* = 0.859, Cochran–Armitage test). No serious ADRs were observed, and all ADRs were mild in severity.

**Table 3:** Incidence of AEs and ADRs

	Placebo (*n* = 20)		Dotinurad							
			1 mg (*n* = 20)		2 mg (*n* = 19)		4 mg (*n* = 21)		Total (*n* = 60)	
	AEs	ADRs	AEs	ADRs	AEs	ADRs	AEs	ADRs	AEs	ADRs
Number of events	16	4	7	3	21	8	18	3	46	14
Number of patients	7	3	6	3	12	5	11	2	29	10
Incidence (%)	35.0	15.0	30.0	15.0	63.2	26.3	52.4	9.5	48.3	16.7
χ^2^ test	P vs. 1 mg	P vs. 2 mg	P vs. 4 mg	1 mg vs. 2 mg	1 mg vs. 4 mg	2 mg vs. 4 mg	P vs. total		Cochran–Armitage test	
*P* value (AEs)	0.736	0.079	0.262	0.038*	0.146	0.491	0.299		0.089	
*P* value (ADRs)	1.000	0.382	0.592	0.382	0.592	0.163	0.861		0.859	

Incidence (%) = Number of patients/Number of analyzed patients × 100

Cochran–Armitage test was used in the 1, 2, and 4 mg, and placebo groups

*P* placebo, *Total* total of dotinurad

*P < 0.05

Gouty arthritis was observed in one (5.0%), one (5.3%), and one (4.8%) patient in the dotinurad 1, 2, and 4 mg groups, respectively, and was not observed in the placebo group (Table 4). No significant differences were observed for any group (*P* ≥ 0.299, χ^2^ test). All gouty arthritis events were judged to be ADRs and mild or moderate in severity at an investigator’s discretion. There was no difference in the onset of gouty arthritis (Table 4).

**Table 4:** Incidence of gouty arthritis

	Placebo (*n* = 20)		Dotinurad					
			1 mg (*n* = 20)		2 mg (*n* = 19)		4 mg (*n* = 21)	Total (*n* = 60)
Number of events	0		1		1		1	3
Number of patients	0		1		1		1	3
Incidence (%)	0.0		5.0		5.3		4.8	5.0
Time points								
Week 2	0		0		0		0	0
Week 4	0		1		1		0	2
Week 6 and Week 8	0		0		0		1	1
χ^2^ test	P vs. 1 mg	P vs. 2 mg	P vs. 4 mg	1 mg vs. 2 mg	1 mg vs. 4 mg	2 mg vs. 4 mg	P vs. total	Cochran–Armitage test
*P* value	0.311	0.299	0.323	0.970	0.972	0.942	0.308	0.446

Incidence (%) = Number of patients/Number of analyzed patients × 100

Cochran–Armitage test was used in the 1, 2, and 4 mg, and placebo groups

*P* placebo, *Total* total of dotinurad

## Discussion

URAT1 is a urate transporter responsible for almost all uric acid reabsorption capacity in the renal tubules, and is the primary target of benzbromarone and lesinurad. Dotinurad, a new selective URAT1 inhibitor, also increases renal uric acid excretion by inhibiting its reabsorption. In the phase 1 study of dotinurad for healthy adult subjects to date, we confirmed that dotinurad reduced serum uric acid levels and amount of urinary uric acid excretion increased in a dose-dependent manner [NCT02348307]. In this study, we investigated the efficacy and safety of dotinurad by recruiting hyperuricemic Japanese patients with or without gout who were given dotinurad for 8 weeks.

In the dotinurad groups, the percent change in serum uric acid level from the baseline to the final visit and the percentage of patients achieving a serum uric acid level ≤ 6.0 mg/dL increased in a dose-dependent manner.

Regarding safety, most AEs were mild or moderate in severity and there were no serious AEs. Additionally, no significant differences were observed in the incidences of AEs among all groups. Gouty arthritis was observed in only one patient in each of the dotinurad group, a finding that was not observed in the placebo group.

Hepatic impairment, such as increased AST or ALT, is often reported as an ADR during treatment of hyperuricemia with or without gout. Benzbromarone has been reported to cause severe hepatic impairment, such as fulminant hepatitis. Therefore, in Japan, treatment with benzbromarone is contraindicated to patients with hepatic impairment. These hepatic toxicities of benzbromarone may be originated from its chemical structure [13]. Because of these fatal problems, benzbromarone is no longer commercially available in the United States and a part of Europe. Regarding the incidence of hepatic impairment-related AEs in this study, increased AST and ALT were observed in 3.3% (two events in two patients, respectively) of the entire dotinurad group, while these events were not observed in the placebo group. Of these cases, the increased ALT observed in the dotinurad 4 mg group was severe in severity, however, elevation of ALT hepatic impairment had already found before starting the study. Consequently, a causal relationship between this event and the study drug was ruled out. Furthermore, there were no significant increases in liver parameters, such as AST, ALT, and γ-GTP, throughout the entire study period in all groups (Table 5).

**Table 5:** Summary of laboratory data

	Placebo			Dotinurad								
				1 mg			2 mg			4 mg		
	*n*	Mean ± SD	*P* value	*n*	Mean ± SD	*P* value	*n*	Mean ± SD	*P* value	*n*	Mean ± SD	*P* value
AST (standard value: 10–40 U/L)												
Run-in period	20	25.9 ± 4.4	–	20	24.7 ± 6.8	–	19	28.1 ± 6.0	–	21	31.6 ± 8.0	–
Week 2	20	24.6 ± 5.5	0.299	20	23.9 ± 7.0	0.247	19	26.9 ± 8.5	0.589	21	32.5 ± 10.2	0.591
Week 4	19	24.0 ± 4.2	0.206	20	24.7 ± 7.6	0.942	19	29.7 ± 19.3	0.657	21	29.4 ± 10.1	0.196
Week 6	19	24.3 ± 4.9	0.347	19	24.9 ± 11.1	0.967	19	26.8 ± 8.6	0.416	21	30.3 ± 10.5	0.480
Week 8	19	24.4 ± 4.9	0.262	18	22.9 ± 5.4	0.095	18	25.6 ± 6.2	0.031*	21	31.6 ± 18.6	1.000
ALT (standard value: 5–45 U/L)												
Run-in period	20	28.2 ± 13.3	–	20	22.4 ± 10.6	–	19	26.0 ± 9.1	–	21	37.1 ± 19.5	–
Week 2	20	25.4 ± 11.3	0.285	20	22.8 ± 10.9	0.658	19	26.3 ± 10.3	0.895	21	35.4 ± 16.1	0.501
Week 4	19	23.5 ± 10.2	0.166	20	24.3 ± 11.9	0.206	19	28.1 ± 21.3	0.664	21	31.9 ± 16.9	0.132
Week 6	19	24.7 ± 10.1	0.284	19	23.2 ± 13.6	0.757	19	26.1 ± 11.7	0.983	21	32.5 ± 15.8	0.083
Week 8	19	23.9 ± 8.9	0.124	18	20.6 ± 9.3	0.179	18	24.3 ± 7.4	0.132	21	43.5 ± 69.2	0.612
γ-GTP (standard value: ≤ 80 U/L)												
Run-in period	20	64.2 ± 49.6	–	20	46.9 ± 39.8	–	19	84.4 ± 102.3	–	21	72.9 ± 46.8	–
Week 2	20	58.1 ± 31.0	0.386	20	46.6 ± 39.4	0.872	19	93.1 ± 136.4	0.356	21	69.2 ± 48.2	0.567
Week 4	19	52.1 ± 27.2	0.253	20	49.0 ± 41.1	0.235	19	95.7 ± 153.7	0.428	21	69.0 ± 46.1	0.572
Week 6	19	49.5 ± 21.5	0.185	19	47.0 ± 43.4	0.234	19	94.3 ± 140.2	0.363	21	68.1 ± 46.9	0.511
Week 8	19	53.6 ± 25.9	0.377	18	40.6 ± 37.0	0.594	18	98.1 ± 148.3	0.403	21	69.0 ± 47.8	0.614
Creatinine (standard value: 0.61–1.04 mg/dL)												
Run-in period	20	0.814 ± 0.090	–	20	0.842 ± 0.084	–	19	0.879 ± 0.096	–	21	0.859 ± 0.096	–
Week 2	20	0.831 ± 0.109	0.357	20	0.856 ± 0.092	0.247	19	0.866 ± 0.075	0.353	21	0.899 ± 0.104	0.031*
Week 4	19	0.838 ± 0.105	0.269	20	0.845 ± 0.100	0.779	19	0.865 ± 0.089	0.468	21	0.871 ± 0.117	0.322
Week 6	19	0.830 ± 0.108	0.387	19	0.832 ± 0.108	0.587	19	0.864 ± 0.089	0.355	21	0.872 ± 0.109	0.373
Week 8	19	0.833 ± 0.113	0.383	18	0.844 ± 0.077	0.963	18	0.863 ± 0.102	0.480	21	0.888 ± 0.116	0.039*

Paired *t *test was used to compare with baseline values

*P < 0.05

Lesinurad was recently approved in combination with XOIs for hyperuricemic patients with gout in the United States and European countries. Though lesinurad has a strong serum uric acid level lowering effect in combination with an XOI, acute renal injury and a serum creatinine increase of ≥ 2.0 times the baseline were observed in a clinical study [7]. In contrast, the present study showed that no AEs associated with renal impairment were observed. Furthermore, regarding the serum creatinine level, no increase of ≥ 2.0 times the baseline was observed. While there were significant increases in mean creatinine level at weeks 2 and 8 in the dotinurad 4 mg group, these mean changes were within the normal physiological fluctuation. Therefore, one can safety conclude that renal function remained stable (Table 5).

Although no significant safety problems were observed in the dotinurad groups in the study, further long-term studies may be needed to obtain additional safety information on dotinurad, hopefully establishing the clinical usefulness of this drug in the treatment of hyperuricemia with or without gout.

In conclusion, dotinurad substantial lowers serum uric acid in a dose dependent fashion in hyperuricemic patients with or without gout. No significant adverse event was found.
